# Effects of a novel onboard sorghum kernel processor and height of cut on berry processing score and ruminal in situ starch disappearance of forage sorghum ensiled for 0 and 90 days

**DOI:** 10.3168/jdsc.2025-0762

**Published:** 2025-06-12

**Authors:** Douglas Duhatschek, Artur Grando Pilati, Lyndon Luckasson, John Goeser, Elizabeth Coons, Luiz F. Ferraretto, Jourdan Bell, Jason K. Smith, Sushil Paudyal, Juan M. Piñeiro

**Affiliations:** 1 Department of Animal Science, Texas A&M University, College Station, TX 77845; 2 Scherer Inc., Sioux Falls, SD 57107; 3 Rock River Laboratory Inc., Watertown, WI 53094; 4 Department of Animal and Dairy Sciences, University of Wisconsin, Madison, WI 53706; 5 Department of Soil and Crop Sciences, Texas A&M University, College Station, TX 77805

## Abstract

The objectives of this experiment were to evaluate the effects of forage sorghum silage harvest settings, combining cut height, onboard sorghum kernel processor (KP) technology, and ensiling duration, on berry processing score (BPS_2.36_) and ruminal in situ starch disappearance at 7 h (isSD7). Three harvest settings were tested: a 20-cm cut height, with and without KP (Low+KP, Low-noKP), and a 120-cm cut height with KP (High+KP). A commercial sorghum field was divided into 9 squares, with 3 squares randomly selected as blocks. Duplicate samples were collected and ensiled in vacuum-sealed bags for 0 or 90 d. Samples were analyzed for nutrient composition, BPS_2.36_, and isSD7. Raising the cut height from 20 to 120 cm increased DM, CP, and starch content, and reduced ADF and NDF. The BPS_2.36_ was lowest for Low-noKP, intermediate for Low+KP, and highest for High+KP (8.16%, 56.2%, and 70.8% ± 1.04% of starch, respectively). The isSD7 was increased for kernel-processed sorghum ensiled for 90 d compared with unprocessed or nonensiled samples. The sorghum KP set at 0.5-mm roll clearance enhanced BPS_2.36_ of sorghum, which considerably improved isSD7 following 90 d of ensiling storage duration.

## Highlights

• Using a sorghum kernel processor set with a 0.5-mm roll gap achieved a BPS_2.36_ > 50%.
• Ensiling enhanced ruminal in situ starch disappearance of kernel-processed sorghum after 7 hours.
• Increasing the cutting height further increased BPS_2.36_.

## Graphical Abstract

**Figure:**
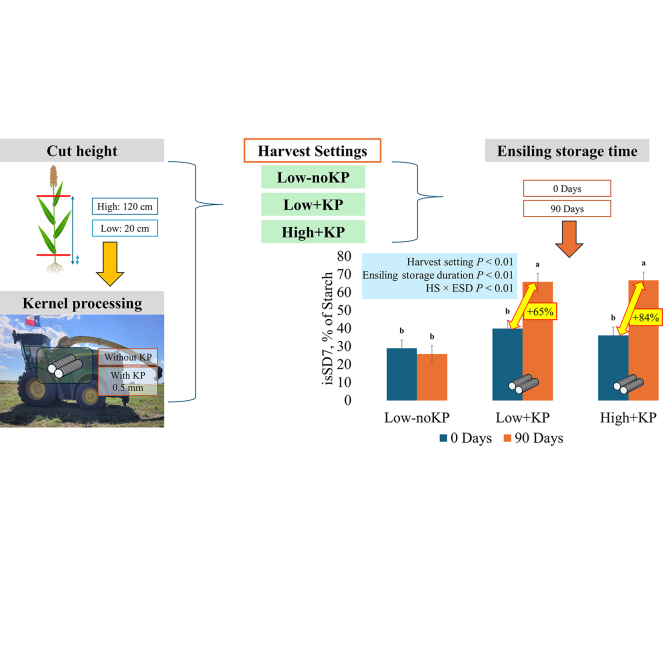
**Summary:** We evaluated the main effects of and interaction between cutting height and the use of a novel sorghum kernel processor (KP) with ensiling storage duration on nutrient composition, berry processing score (BPS_2.36_), and in situ starch disappearance of forage sorghum. Increasing cutting height increased dry matter, crude protein, and starch contents and decreased acid detergent fiber and neutral detergent fiber. The use of the new sorghum kernel processor with 0.5-mm roll gap improved BPS_2.36_ by 7- and 10-fold for low and high cut heights, respectively. Ensiling kernel-processed sorghum further enhanced in situ starch disappearance compared with unprocessed ensiled or nonensiled sorghum.

Starch digestibility of whole-plant sorghum silage is typically lower than corn silage. Compared with corn, sorghum starch digestibility was suggested to be lower because of the greater proportion of peripheral endosperm and the lower solubility of the main protein component of the protein starch matrix, which is kafirin in sorghum and zein in corn grains (Rooney and Pflugfelder, 1986; McCary, 2019). The peripheral endosperm is dense and resistant to water penetration and digestion (Rooney and Pflugfelder, 1986). The ruminal in situ starch disappearance at 7 h (**isSD7**) of sorghum silages in the west region of the United States in 2024 was approximately half of the isSD7 of corn silage in the same area according to the Rock River Laboratory nutrient statistics report (42.3% ± 16.0% vs. 84.1% ± 12.3% of starch, respectively, median ± SD; Rock River Laboratory Database, 2024). Although protein solubility differences partly explain this disparity, other factors such as grain processing, maturity, genetics, and ensiling storage duration also play crucial roles (Ferreira and Mertens, 2005; Dias Junior et al., 2016; McCary, 2019).

Grain processing is a key determinant factor of starch digestibility. Sorghum, like corn grain, has an outer layer named pericarp that functions as a physical barrier protecting the inner components of the grain from microbial and enzymatic attack, therefore inhibiting its fermentation and digestion (Huntington, 1997). Grain processing disrupts the pericarp and increases the surface area of grain particles. Dias Junior et al. (2016) demonstrated that increasing the degree of corn grain processing increased the surface area of corn grain particles from 3.6 cm^2^/g in whole grain to 23.2 cm^2^/g when grain was cut into 64 pieces. Furthermore, increasing corn grain processing increased ruminal DM disappearance at 6 h from 4.5% to 34.6% ± 6.1% of DM for whole versus grain cut into 64 pieces (Dias Junior et al., 2016). Additionally, decreasing mean particle size linearly increased the effective rumen starch degradability of corn grain (Rémond et al., 2004). In sorghum, McCary (2019) observed an increase in effective ruminal disappearance of starch from 15.2% to 39.7% when whole sorghum berries were compared with berries cut into 4 pieces. Additionally, Shipandeni et al. (2023) compared dry corn and sorghum grains milled through 1- or 4-mm screens (fine and coarse, respectively) and reported increased in vitro starch disappearance after 7 h of incubation for both grains when finely ground.

To evaluate the extent of grain processing in corn silage, Ferreira and Mertens (2005) developed the corn silage kernel processing score. This score is defined as the proportion of starch passing through a 4.75-mm sieve because only kernel fragments of one-fourth of whole corn kernel or smaller passed through this sieve (Ferreira and Mertens, 2005). In sorghum, because of the smaller berry size compared with corn, Johnson (2017) suggested the use of the proportion of starch passing the 1.70-mm sieve to assess sorghum berry processing (**BPS_1.70_**) as an alternative to kernel processing score. However, McCary (2019) found that 73.9% and 14.1% of quartered sorghum berries were retained above the 1.70- and 2.36-mm sieves, respectively, indicating that the use of BPS_1.70_ could underestimate sorghum berry processing. Thus, McCary (2019) proposed the proportion of starch passing through a 2.36-mm sieve as a revised berry processing score (**BPS_2.36_**). For corn silage, the industry is consistently achieving appropriate grain processing. The kernel processing score in the United States has been above 65% since 2019 and was 71.1% in 2024 (Karlen and Goeser, 2024). However, sorghum silage berry processing has historically been suboptimal. Raver et al. (2022) reported that 85% of sorghum silage samples surveyed in the United States in 2022 had BPS_2.36_ <30%. Thus, optimizing berry processing when harvesting sorghum silage represents a large opportunity to increase overall starch digestibility.

The smaller berry size of sorghum contributes to greater processing difficulty during harvest compared with corn. While 100% of intact whole corn kernels were retained above the 6.25-mm sieve, 0% of intact sorghum grain was retained on the same sieve and approximately 97% of intact sorghum berries were retained above the 2.36-mm sieve (Dias Junior et al., 2016; McCary, 2019). In an attempt to increase BPS_2.36_, McCary (2019) tested a short and long (15 and 22 mm) theoretical length of cut (**TLC**) with a conventional kernel processor (**KP**) set at a 1- or 3-mm clearance gap between rolls when harvesting sorghum. The study concluded that harvesting sorghum with a short TLC combined with a 1-mm KP clearance gap increased BPS_2.36_ when compared with the other harvest setting combinations. However, the effective ruminal disappearance of starch was only marginally improved for short TLC with a 1-mm KP clearance gap when compared with short TLC and 3-mm KP clearance gap but was not different from long TLC with either a 1- or 3-mm KP clearance gap (McCary, 2019). These results indicate that aggressive processing at harvest can increase sorghum silage BPS_2.36_. However, regardless of harvest setting, further improvements in ruminal starch disappearance may require specialized harvesting equipment.

To address these challenges, an experiment was conducted to evaluate a novel onboard KP (Durracut, Scherer Inc., Sioux Falls, SD) specifically designed to improve berry processing of sorghum silage. We hypothesized that harvesting forage sorghum with this KP would increase BPS_2.36_ compared with harvesting without a KP. Furthermore, we hypothesized that ensiling for 90 d adequately processed sorghum—harvested using this KP—would enhance isSD7 when compared with unprocessed or nonensiled material.

The objective of this randomized complete block design was to assess the effect of 3 harvest settings (**HS**) and 2 ensiling storage durations (**ESD**) on sorghum BPS_2.36_ and isSD7. A forage sorghum (Pearl, Mojo Seed, Hereford, TX) grown under central pivot irrigation was harvested at the hard dough stage at a single production site in Clovis, New Mexico. The field was initially divided into 9 squares. Three of the 9 squares were randomly selected and used as blocking factors to control field variation. Each of the 3 HS were randomly applied within each square and within approximately 100 m in the same harvest row. The HS tested were low cut height without KP (**Low-noKP**), low cut height with sorghum KP (**Low+KP**), and high cut height with sorghum KP (**High+KP**). Because this experiment was conducted on a commercial field, we were unable to include a fourth treatment (i.e., high cut without KP). Low cut height was harvested approximately 20 cm above the ground and represented whole-plant silage, whereas the high cut height was harvested approximately 120 cm above the ground and represented headlage. The KP was operated at 50% speed differential and was set with a 0.5-mm clearance gap between the rolls. The TLC was 16 mm.

Duplicate samples from each HS on each square were collected and placed into vacuum-sealed laboratory silo bags composed of multilayer, nylon-reinforced plastic, which were sealed using a vacuum sealer with heat sealing function. Bags were stored for 0 or 90 d to assess the effect of ESD on BPS_2.36_ and isSD7. Silo bags contained approximately 0.5 kg of fresh forage and were stored in a room with temperature at 20°C. Particle size distribution of fresh samples was assessed with the Penn State Particle Separator as described by Kononoff et al. (2003). After ensiling for 0 or 90 d, each laboratory silo was split into 2 subsamples. One set of subsamples were sent to a commercial laboratory (Rock River Laboratory, Watertown, WI) for nutrient composition and berry processing score analysis. Dry matter was assessed by drying the samples in a forced ventilation oven set to 55°C until constant weight. Dry samples were ground to 1 mm (Cyclone sample mill, UDY Corporation, Fort Collins, CO) and analyzed by near-infrared spectroscopy (**NIRS**) using a Foss 5000 (Foss North America Inc., Eden Prairie, MN). The calibration equations for NIRS analysis of starch, NDF, ADF, and CP were based on the following wet chemistry procedures: enzymatic reaction for starch (modified AOAC 2014.10; Hall et al., 2015); Dumas method for CP (AOAC International, 2005); sequential detergent fiber analyses were used to determine NDF and ADF concentration on an Ankom fiber analyzer (adapted from Van Soest et al., 1991). Berry processing score was defined as the percentage of starch passing through the 2.36-mm sieve (**BPS_2.36_**) as described by McCary (2019).

The other set of subsamples was sent to the University of Wisconsin (Madison, WI) where it was assayed for ruminal in situ starch disappearance at 0 and 7 h. Ruminal in situ incubations were conducted under an approved protocol by the Animal Care and Use Committee of the College of Agriculture and Life Sciences at the University of Wisconsin–Madison. Two mid-lactation multiparous Holstein cows fed a diet containing approximately 25% starch and DM ingredient proportions of 21% corn silage, 30.5% alfalfa silage, 42.7% concentrate, and 5.8% whole cottonseed were used for ruminal in situ incubations. Undried and unground samples were used to maintain the effects of berry processing on ruminal in situ starch disappearance. Triplicate bags containing approximately 5.00 ± 0.28 g of DM of each sample were placed into polyester bags (10 × 20 cm, 50 ± 10 µm porosity; R1020 Ankom Technologies). Each sample was incubated in the ventral sac of the rumen for 0 and 7 h in duplicates within each cow. After removal, sample bags were submerged in cold water (water + ice) for 15 min and rinsed with room temperature tap water to wash off any large particles adhered to the bags. Sample bags were then placed in clean laundry bags for further washing in a washing machine set on the rinse and spin cycle with room temperature water for 30 min. Dry residue from each sample within cow was combined and ground to pass a 1-mm sieve in a Cyclone mill. The ground residue was then analyzed for starch concentration by an enzymatic method (Hall et al., 2015) with thermostable α-amylase (Ankom Technology, Macedon, NY) and amyloglucosidase (Megazyme E-AMGDF, Bray, Co. Wicklow, Ireland) enzymes. Fraction A was defined as starch immediately degraded upon ruminal incubation for 0 h.

Statistical analysis was performed with the Glimmix and Corr procedures of SAS (SAS version 9.4, SAS Institute Inc., Cary, NC). Mixed linear regression analyses included HS, ESD, and their interactions as main factors in all models except for particle size distribution where only HS was included as a fixed factor. In addition, blocks were the random factor in all models. The *P*-values were adjusted using Bonferroni correction for multiple comparisons. Assumptions of normality of residuals and homoscedasticity were confirmed through the visual inspection of the Q-Q plots and residual scatterplots in combination with the Shapiro-Wilk test where all samples yielded a W ≥0.95. Statistical significance was considered when *P* < 0.05.

Harvest settings affected forage particle size distribution (Table 1), and the use of sorghum KP increased forage processing. Both HS treatments that included sorghum KP decreased the proportion of particles retained on the 19-mm screen of the Penn State Particle Separator (*P* < 0.0001). Our findings align with those of Cooke and Bernard (2005), who reported that harvesting corn silage with a 2-mm roll gap decreased the proportion of particles retained on the 19-mm screen compared with the use of KP with 8-mm clearance gap or not utilizing KP. In a review, it was concluded that at the same TLC, the use of KP decreases the proportion of particles retained on the 19-mm sieve by 20% when harvesting whole-plant corn silage (Ferraretto et al., 2018). Furthermore, the proportion of particles between the 19- and 8-mm sieves was less for the High+KP compared with Low+KP and Low-noKP (*P* < 0.01). This is probably because of the reduced proportion of stem and leaves harvested due to increased cutting height from the High+KP treatment.

**Table 1:** Nutrient composition, berry processing score, starch degradability, and particle size distribution of forage sorghum harvested with low cut height with and without kernel processor (Low+KP and Low-noKP), and high cut height with sorghum kernel processor (High+KP) and ensiled for 0 or 90 d

Item	Low-noKP		Low+KP		High+KP		SEM	*P-*value^1^		
	0 d	90 d	0 d	90 d	0 d	90 d		HS	ESD	HS × ESD
DM, % of total	37.6	37.2	39.2	40.0	56.4	56.9	1.39	<0.0001	0.74	0.85
CP, % of DM	9.58	9.38	9.93	9.73	11.8	11.5	0.46	0.001	0.55	0.99
ADF, % of DM	24.3	23.9	22.1	22.0	15.9	16.7	1.13	<0.0001	0.91	0.85
NDF, % of DM	36.1	35.5	32.7	29.8	23.4	23.9	1.15	<0.0001	0.25	0.29
Starch, % of DM	33.1	30.6	37.2	36.7	52.1	51.2	1.67	<0.0001	0.29	0.76
BPS_2.36_,^2^ %	7.71	8.61	55.8	56.7	70.0	71.6	1.28	<0.0001	0.22	0.93
Fraction A, % of starch	6.40	8.49	19.5	23.0	17.3	31.8	5.29	0.01	0.11	0.36
isSD7,^3^ % of starch	29.0^b^	25.8^b^	39.9^b^	65.9^a^	36.2^b^	66.7^a^	4.62	0.0002	0.0005	0.007
Ammonia-N, % of CP	6.02	10.4	7.62	12.3	10.7	13.9	0.40	<0.0001	<0.0001	0.18
Soluble CP, % of CP	35.4	48.9	32.3	44.8	25.6	48.9	1.50	<0.0001	<0.0001	0.13
Particle size distribution,^4^ % of total original matter										
>19 mm	12.5	—	3.10	—	1.82	—	0.78	<0.0001	—	—
8–19 mm	56.0	—	50.2	—	23.4	—	2.29	<0.0001	—	—
4–8 mm	24.7	—	26.0	—	31.1	—	2.43	0.23	—	—
Pan	6.81	—	20.8	—	43.7	—	1.48	<0.0001	—	—

a,b Within a row, means with different superscripts differ at *P* < 0.05 for the interaction of HS × ESD.

1 Main effects of harvest settings (HS; Low-noKP, Low+KP, or High+KP) and ensiling storage duration (ESD; 0 or 90 d).

2 Berry processing score, represented as the percentage of starch passing the 2.36-mm sieve (McCary, 2019).

3 In situ ruminal starch disappearance at 7 h.

4 Particle size distribution of fresh samples assessed with the Penn State Particle Separator as described by Kononoff et al. (2003).

Harvest settings altered nutrient composition of sorghum silage. The NDF and ADF were lowest, whereas starch and CP were highest for High+KP (*P* < 0.001, *P* < 0.0001, *P* < 0.0001, *P* = 0.001, respectively). Ferraretto et al. (2018) reviewed the literature and found increased starch and CP content when whole-plant corn silage was compared with snaplage. In addition, NDF content was decreased for snaplage when compared with whole-plant corn silage. These changes were attributed to the difference in proportion of grain to stover when harvesting whole-plant silage or snaplage. Similarly, in the present experiment, increasing the height of cut decreased the proportion of stover harvested, decreasing structural carbohydrates and increasing starch and CP from grain. The greater starch content in Low+KP compared with Low-noKP was not expected and may be the result of a combination of sampling error, field variation, or analytical error.

Including the sorghum KP technology increased BPS_2.36_ of sorghum silage compared with unprocessed silage. Berry processing score was greater for High+KP, intermediate for Low+KP, and lowest for Low-noKP (*P* < 0.0001). Although BPS_2.36_ was greater for High+KP than Low+KP, both were above the targeted 50% starch passing the 2.36-mm sieve. The use of KP has been proven to decrease the particle size of corn kernels in whole-plant corn silage, as narrowing the roll gap between the KP rolls from 5 to 2 mm lowered the mean particle size of corn kernels from 2.4 to 0.8 mm (Ferraretto et al., 2018). In sorghum, however, decreasing the KP roll gap from 3 to 1 mm increased BPS_2.36_ only when combined with a shorter TLC (McCary, 2019). In the present study, the use of sorghum KP with a 0.5-mm roll gap resulted in BPS_2.36_ above 50% regardless of cutting height. Additionally, berry processing was further enhanced by increasing the cut height (High+KP vs. Low+KP). This outcome likely reflects both the narrower KP roll gap and the specialized design of the KP in our experiment. Moreover, higher cut height can decrease the proportion of stover to grain entering the KP and potentially increase grain processing by the forage harvester knives and KP, thereby further improving berry processing (Mertens, 2005).

Ensiling increased soluble CP and ammonia-N (*P* < 0.0001 and *P* < 0.0001, respectively). Proteolytic activity of enzymes and microbes during the ESD are well established in the literature (Hoffman et al., 2011; Der Bedrosian et al., 2012; Ferraretto et al., 2014). Furthermore, ammonia-N was greatest for High+KP and lowest for Low-noKP (*P* < 0.0001). Narrowing the KP roll gap decreases particle size and may increase surface area for microbial enzymatic activity, which includes deamination of AA (Hoffman et al., 2011). Similarly, Saylor et al. (2021a) found a greater concentration of ammonia-N when corn silage was processed with a KP roll gap of 1 mm compared with 3 mm. Moreover, the authors reported increased soluble CP for corn silage harvested at a later stage of maturity when compared with early maturity at 120 d of ESD, whereas no significant differences were observed at other ESD (Saylor et al., 2021a). In the current experiment, soluble CP was greatest for Low-noKP, intermediate for Low+KP, and greatest for High+KP (*P* < 0.0001). This effect was likely due to increased moisture contents of the low cut treatments that potentially allowed for a more robust fermentation (Kung et al., 2018).

The increased berry processing observed in the High+KP and Low+KP increased fraction A of starch compared with Low-noKP (*P* = 0.01). Fraction A of starch was shown to increase with decreased particle size of dry ground corn (Rémond et al., 2004) and when sorghum berries were quartered when compared with whole or halved berries (McCary, 2019). In contrast, harvesting whole-plant sorghum silage with a 1-mm compared with 3-mm KP roll gap clearance using conventional KP, or short compared with long TLC, did not significantly affect fraction A of starch (*P* = 0.10; McCary, 2019).

There was an interaction between HS and ESD on isSD7. In situ starch disappearance at 7 h increased with prolonged ensiling (0 to 90 d) for sorghum harvested with KP (Low+KP and High+KP), whereas no change was observed for sorghum harvested without KP (Low-noKP). Increasing the ESD extends the stable phase of the silage-making process, allowing more time for proteolysis and solubilization of cross-linked prolamins in the protein matrix surrounding the starch granules (Hoffman et al., 2011; Der Bedrosian et al., 2012), thus increasing the availability of starch granules embedded in the protein matrix and enhancing starch digestibility (Hoffman et al., 2011; Der Bedrosian et al., 2012; Ferraretto et al., 2014). However, in the Low-noKP treatment, poor grain processing likely limited pericarp disruption, restricting access to starch granules and reducing the effectiveness of this process.

The benefits of prolonged ESD on isSD7 may require exposure of the grain endosperm through the disruption of the pericarp. Saylor et al. (2021b) ensiled corn silage with intact or damaged grain pericarp and found that ESD improved starch digestibility only when the pericarp was damaged. Similarly, McCary (2019) observed a linear increase in the digestibility rate of fraction B of starch when sorghum harvested with KP roll gap of 1 mm was ensiled for 0, 30, and 90 d, whereas this effect was not observed when sorghum was harvested with KP roll gap of 3 mm. Similarly, improvements in isSD7 with 90 d of ESD when silage was harvested without KP were not observed in the current experiment. Moreover, the linear correlation of starch digestibility with soluble CP and ammonia-N previously reported by other authors (Der Bedrosian et al., 2012; Ferraretto et al., 2014) was not observed for Low-noKP in this experiment, whereas it existed for HS that included sorghum KP (Figure 1). These results support the notion that the intact pericarp protects the grain endosperm from proteolytic activity of enzymes and microbes, and suggest that berry processing before ensiling is critical to expose grain endosperm. Such exposure allows for more extensive proteolysis of the protein matrix surrounding the starch granules during prolonged ESD, enhancing isSD7 for ensiled sorghum silage compared with nonensiled kernel-processed or unprocessed sorghum silage.

**Figure 1:**
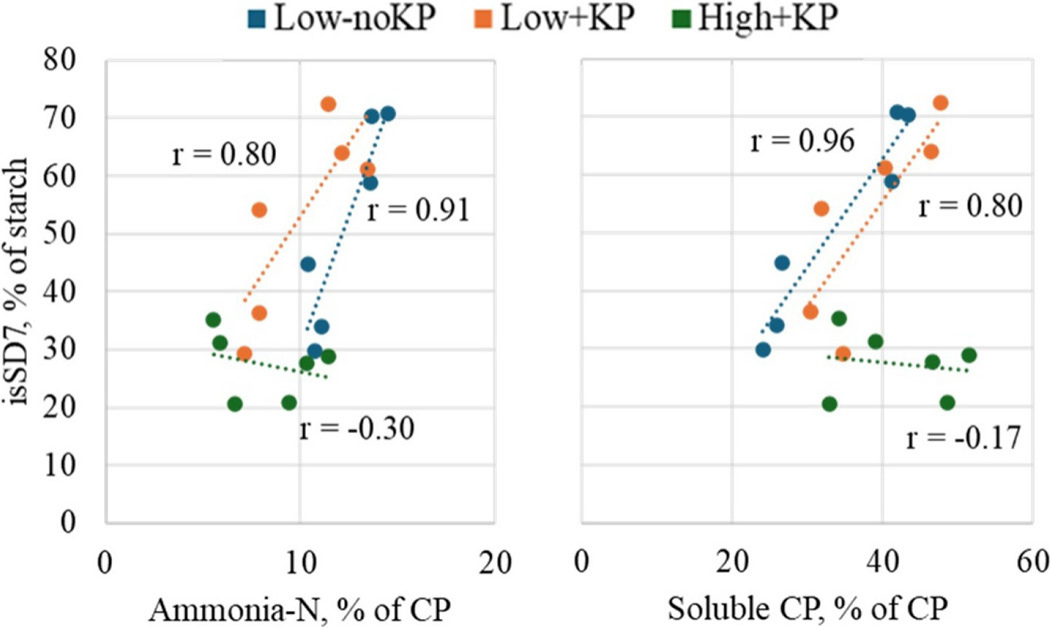
Correlation between ruminal in situ starch disappearance at 7 h (isSD7) with ammonia-N (A) and soluble CP (B) of forage sorghum harvested with low cut height without KP (Low-noKP), low cut height with sorghum KP (Low+KP), and high cut height with sorghum KP (High+KP). Positive correlations were observed for High+KP with both ammonia-N (*P* = 0.01) and soluble CP (*P* = 0.002); Low+KP showed trends toward significance for both variables (*P* = 0.05), whereas no significant correlations were found for Low-noKP (*P* = 0.57 and 0.75, respectively).

The results of the present study suggest that ESD increases isSD7 of forage sorghum harvested with sorghum KP compared with unprocessed or nonensiled sorghum. In addition, utilizing sorghum KP technology set with 0.5-mm roll gap resulted in BPS_2.36_ >50% and improved the fraction A digestion of starch, regardless of cutting height. Increasing the cutting height improved starch content and further increased BPS_2.36_, likely due to reduced stover intake during harvest. However, the additional increase in BPS_2.36_ did not further enhance isSD7.
