# ER to: Effects of a novel onboard sorghum kernel processor and height of cut on berry processing score and ruminal in situ starch disappearance

**DOI:** 10.3168/jdsc.2026-7-4-698

**Published:** 2026-08-31

**Authors:** Douglas Duhatschek, Artur Grando Pilati, Lyndon Luckasson, John Goeser, Elizabeth Coons, Luiz F. Ferraretto, Jourdan Bell, Jason K. Smith, Sushil Paudyal, Juan M. Piñeiro

**Affiliations:** 1 Department of Animal Science, Texas A&M University, College Station, TX 77845; 2 Scherer Inc., Sioux Falls, SD 57107; 3 Rock River Laboratory Inc., Watertown, WI 53094; 4 Department of Animal and Dairy Sciences, University of Wisconsin, Madison, WI 53706; 5 Department of Soil and Crop Sciences, Texas A&M University, College Station, TX 77805

This article contained a treatment-labeling error in Figure 1, in which the legends for High+KP and Low-noKP were switched. The corrected figure appears below.

**Figure 1:**
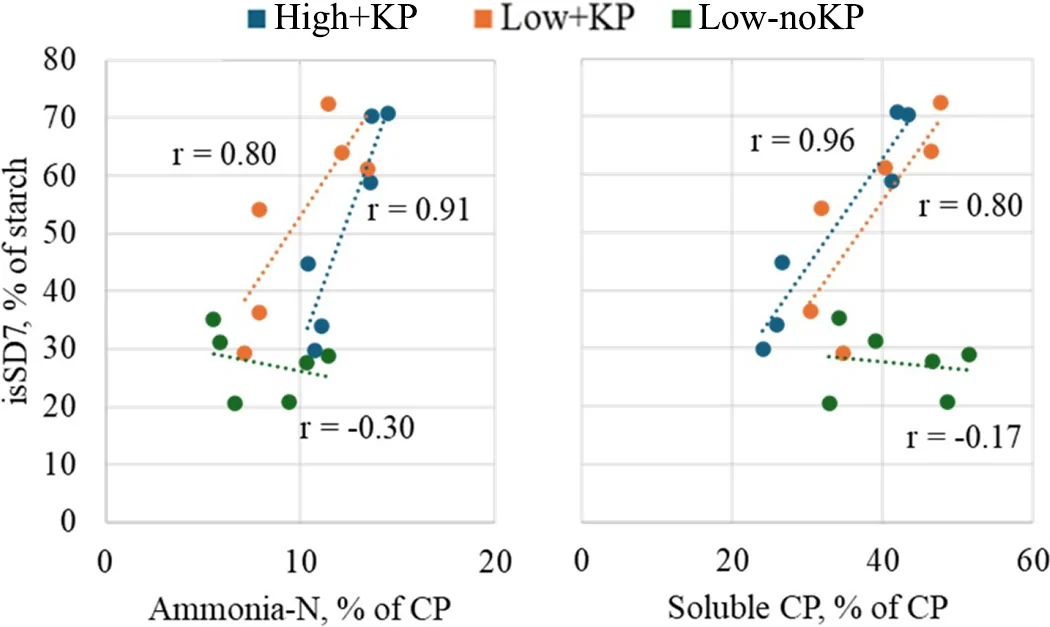
Correlation between ruminal in situ starch disappearance at 7 h (isSD7) with ammonia-N (A) and soluble CP (B) of forage sorghum harvested with low cut height without KP (Low-noKP), low cut height with sorghum KP (Low+KP), and high cut height with sorghum KP (High+KP). Positive correlations were observed for High+KP with both ammonia-N (*P* = 0.01) and soluble CP (*P* = 0.002); Low+KP showed trends toward significance for both variables (*P* = 0.05), whereas no significant correlations were found for Low-noKP (*P* = 0.57 and 0.75, respectively).

The authors regret the error.
